# Lichens are a treasure chest of bioactive compounds: fact or fake?

**DOI:** 10.1111/nph.70034

**Published:** 2025-02-27

**Authors:** Anna Pasinato, Garima Singh

**Affiliations:** ^1^ Department of Biology University of Padova Via U. Bassi, 58/B 35121 Padova Italy; ^2^ National Biodiversity Future Center (NBFC) Piazza Marina, 61 90133 Palermo Italy; ^3^ Botanical Garden University of Padova Via Orto Botanico, 15 35123 Padova Italy

**Keywords:** biosynthetic genes, genome mining, Lecanoromycetes, lichenized fungi, natural products, polyketide synthases, secondary metabolites, symbiotic fungi

## Disclaimer

The New Phytologist Foundation remains neutral with regard to jurisdictional claims in maps and in any institutional affiliations.

## Introduction

Lichenized fungi are regarded as a treasure chest of natural products. This idea stems from the variety of metabolites detected in the extracts of a single lichen (Türk *et al*., 2006; Le Pogam *et al*., 2016a,b; Singh *et al*., 2022). However, the notion that lichens are a treasure chest of secondary metabolites as compared to the non‐lichenized fungi (nLFF) has never been systematically tested.

Lichens are symbiotic associations consisting of a fungal partner in an obligate symbiotic association with one or more photosynthetic partners, such as algae, cyanobacteria, or both (Honegger, 1998, 2023; DePriest, 2004; Grube, 2024). Over time, their definition has expanded to include the associated microbiota, such as yeasts, bacteria, and other life forms, collectively forming the lichen holobiont (Hawksworth & Grube, 2020; Allen & Lendemer, 2022; Cometto *et al*., 2022; Lücking & Spribille, 2024).

Overall, *c*. 1000 metabolites have been characterized from lichens, many of which are bioactive (Huneck & Schreiber, 1972; Huneck & Yoshimura, 1996a,b; Boustie & Grube, 2005; Ranković, 2015; Emsen *et al*., 2017; Calcott *et al*., 2018). While initially whole lichen extracts were tested for bioactivity, recent studies have focused on isolating individual lichen metabolites and evaluating their bioactivity (Ebrahim *et al*., 2016; Ranković & Kosanić, 2019; Ingelfinger *et al*., 2020; Poulsen‐Silva *et al*., 2025). Furthermore, a recent review article highlighted that fine‐scale analyses, such as mass spectrometry, reveal a plethora of unidentified metabolites (Singh *et al*., 2025).

Comparing the metabolic potential of fungi can be challenging as the secreted metabolome of organisms depends on the combination of biotic and abiotic factors, as well as life‐stage‐dependent cues. Secondary metabolites are secreted by a set of dedicated genes called biosynthetic genes, organized in a collinear fashion, resulting in metabolic or biosynthetic gene clusters (BGCs) (Firn & Jones, 2000; Keller *et al*., 2005; Devashree *et al*., 2021). Depending on the structure of the metabolite coded, BGCs can be classified as polyketide synthase (PKS), nonribosomal peptide synthetase (NRPS), terpene, or ribosomally synthesized and post‐translationally modified peptides (RiPP) cluster (Arnison *et al*., 2013; Helaly *et al*., 2018; Gill *et al*., 2023). These clusters constitute the majority of the BGC landscape of organisms, and a minor portion is also contributed by indoles, isocyanide synthase, and PKS–NRPS hybrid clusters (Nickles *et al*., 2023). The same fungi, under different life stages and in reaction to different cues, activate different sets of metabolic genes (Keller, 2019). Consequently, organisms possess a broader chemical potential than evident in the secreted metabolome at a particular time (Machado *et al*., 2017; Calcott *et al*., 2018; Gavriilidou *et al*., 2022). Furthermore, studies show that silent BGCs can be activated, leading to the production of metabolites. These facts imply that BGC content is a better indicator of the metabolic potential of fungi. (Fujii *et al*., 1996; Bok *et al*., 2006; Guo & Wang, 2014; Chen *et al*., 2017; Miethke *et al*., 2021; Navarro‐Muñoz & Collemare, 2022).

Here, we assess whether lichens are a treasure trove of natural products using the total number of BGCs as a proxy for their biochemical potential and comparing this metric between LFF and nLFF. Additionally, we investigate whether certain BGC classes are specifically enriched in either group.

## Materials and Methods

### Dataset

Most of the lichen biomass is constituted by the lichen‐forming fungus or LFF also known as the mycobiont. The name of a lichen corresponds to the name of the fungal partner (Will‐wolf *et al*., 2004; Lutzoni & Miadlikowska, 2009), which is also the major contributor to its metabolite profile. The lichenized lifestyle is predominantly found in Ascomycota, with over 99% of lichens belonging to this phylum (Lutzoni *et al*., 2004; Cousin, 2014; Gueidan *et al*., 2015; Lücking & Nelsen, 2018). Lichens represent an ancient and classic example of symbiosis, and within Ascomycota, the classes Lecanoromycetes, Arthoniomycetes, and Lichinomycetes are almost exclusively lichenized. Among these, Lecanoromycetes representing the largest clade of LFF, comprising *c*. 78% of all lichens. In addition to these, the lichenized lifestyle is also found in the Ascomycota classes Eurotiomycetes and Dothideomycetes, which are composed of both LFF and nLFF.

A total of 373 ingroup fungal species from Pezizomycotina were included in the study, belonging to nine taxonomic classes in Ascomycota and comprising 136 LFF and 237 nLFF (Supporting Information Table S1). Twenty‐eight taxa from Basidiomycota were included as outgroup. All genomes (or SRA data) are available from the NCBI (https://www.ncbi.nlm.nih.gov/home/genomes/; https://www.ncbi.nlm.nih.gov/sra), and the voucher information of all the genomes, including the accession numbers, is provided in Table S1. Given that LFF exist in symbiotic association and the genomes submitted to NCBI may represent lichen metagenomes, all genomes were binned to extract the fungal reads. Specifically, diamond BLASTx was used, implementing the more‐sensitive mode for longer sequences and a default e‐value cutoff of 0.001 against a custom NCBI GenBank protein database nr (downloaded May 2017) to generate alignments of lichen genomes. The Diamond results were then parsed in Megan v.6 (Huson *et al*., 2016) using max expected set to 1E‐10 and the weighted lowest common ancestor algorithm. All contigs assigned to Ascomycota were exported to represent the mycobiont or the fungal component of the lichen. Assemblathon v.2 was used to estimate the assembly metrics, including the number of contigs and total length (Table S1).

Functional annotation of the genomes, including genes and protein predictions, was performed using the funannotate pipeline (Palmer, 2020). The genomes were first masked for repetitive elements, followed by gene prediction using Busco2 (Benchmarking Universal Single Copy Orthologs) to train Augustus and self‐training GeneMark‐ES (Borodovsky & Lomsadze, 2011). The functional annotation of the predicted genes was done with InterProScan (Blum *et al*., 2021) and egg‐NOG‐mapper (Huerta‐Cepas *et al*., 2019).

### Genome binning and BGC detection

We focus exclusively on the biosynthetic potential of the mycobionts, excluding associated microorganisms (such as bacteria and photobionts). Mycobiont bins were used for identifying BGCs using FungiSMASH (Blin *et al*., 2023), which predicts and annotates the biosynthetic gene cluaters in fungal genomes (antibiotics & SM Analysis Shell, v.7.0 (Blin *et al*., 2023)). Since fungiSMASH predicts BGCs only for the largest 1000 scaffolds, we split fungal genomes into sets of 1000 scaffolds when the total scaffold count exceeded this limit. We categorized the BGCs into following five classes based on the core gene present in them: PKS, NRPS, terpene, RiPP, and hybrid clusters. The number of total BGCs and that of each BGC class were estimated for all the genomes from the fungiSMASH runs.

### Phylogenomic tree

Genome completeness for all the assemblies was inferred using the Benchmarking Universal Single‐Copy Orthologs (BUSCO) pipeline with the Ascomycota database (odb_10) (Simão *et al*., 2015; Manni *et al*., 2021). This pipeline performs a quality assessment of genome assemblies by identifying the presence of conserved orthologous genes and categorizes them as present in single copy, duplicated, fragmented, or missing, based on comparison to a reference set of orthologs present in the chosen reference database, Ascomycota odb_10 in our study. Only genomes with completeness above 90% were analyzed for BGCs to ensure that incomplete assemblies did not lead to misrepresentation of biosynthetic potential.

Single‐copy BUSCOs from 401 taxa (373 ingroup and 28 outgroup taxa) were used for generating the phylogenomic tree. For each taxon, the single‐copy BUSCOs were concatenated, and the concatenated sequences from all the taxa were then aligned using Mafft L‐INS‐i. Evolutionary relationships were inferred from the multiple gene alignment using maximum likelihood analysis implemented in IQTree v1.5.5 with standard model selection and 1000 bootstrap replicates. The resulting tree was visualized using FigTree 1.3.1 (Rambaut, 2010) and annotated in iTOL (Letunic & Bork, 2021).

### Genome quality assessment

For the genomes that were highly fragmented (> 1000 scaffolds), we tested the correlation between the number of scaffolds and the number of BGCs to test whether fragmented genomes lead to an inflated number of BGCs. We also tested the correlation between genome size and the number of BGCs (to infer whether the larger genomes contained a greater number of BGCs). Pearson's product–moment correlation was calculated using the stats (v.3.6.2) R package (R Core Team, 2020, 2022). A correlation coefficient close to 0 suggests no correlation between the variables, while a value nearing 1 indicates a strong positive correlation (Notes S1).

### Statistical assessment of BGCs between lichenized and nonlichenized fungi

To test whether the number of BGCs is significantly higher in LFF, we categorized the data into two broad categories – LFF and nLFF ‐ using lifestyle as criterion, irrespective of the taxonomic relatedness. We then performed ANOVA test using the package *dyplr* in R for the total number of BGCs and for each BGC class.

To account for sampling and data bias, we performed a randomization analysis. For each of the 10 000 iterations, we randomly sampled datasets of 100 LFF and 100 nLFF and conducted a t‐test to assess the statistical significance of differences in BGC counts between the two groups. To investigate specific BGC class enrichment in lichen genomes, we assessed whether the number of PKS, NRPS, terpene, RiPP, and hybrid BGCs were significantly different between LFF and nLFF, using a randomization test with 10 000 iterations and a sample size of 100 taxa per group (Notes S1).

## Results

### Phylogenomic tree

**Fig. 1:**
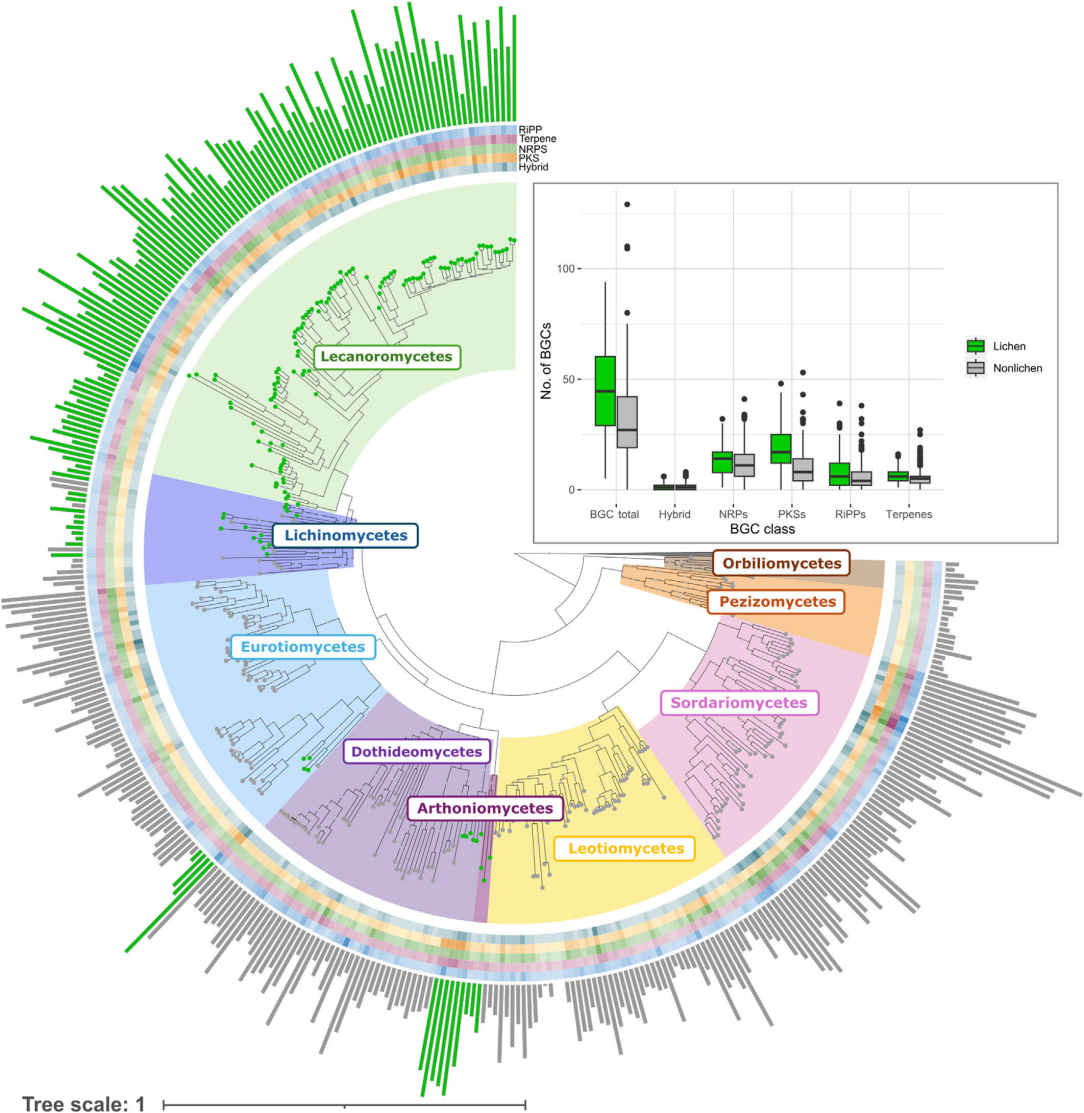
Phylogenomic tree inferred from 401 fungal genomes (1098 single‐copy BUSCOs (Benchmarking Universal Single‐Copy Orthologs)) belonging to nine classes in Pezizomycotina. Lichenized fungal (LFF) genomes are represented by green dots, and non‐lichenized fungal (nLFF) genomes are represented by gray dots at the end of the tree branches. The heatmaps on the outside of the tree show the number of biosynthetic gene clusters (BGCs) of each class (PKS, polyketide synthase; NRPS, nonribosomal peptide synthase; RiPP, ribosomally synthesized and post‐translationally modified peptides, terpene synthases, and PKS–NRPS hybrids) per taxon. The number of total BGCs for each taxon is represented by colored bars (green for LFF, gray for nLFF). In general, LFF have a higher number of BGCs as compared to closely related nLFF. Boxplot shows the differences in the number of BGCs between LFF (green boxes) and nLFF (gray boxes). LFF genomes contain more BGCs than those of nLFF and a significantly higher number of PKS clusters. Horizontal lines on the boxplot represent median, whiskers represent the data out of the interquartile range and the dots represent the outliers.

The phylogenomic species tree, constructed from the alignment of 1098 genes from 401 taxa (600 247 base pairs), was well‐supported (Fig. 1). All taxa previously classified as Lecanoromycetes formed a supported monophyletic clade. Likewise, Eurotiomycetes, Dothideomycetes and Sordariomycetes, and Leotiomycetes also resulted in supported monophyletic clades (Díaz‐Escandón *et al*., 2022; Singh *et al*., 2022; Nickles *et al*., 2023).

### BGC enrichment between lichenized and nonlichenized fungi

FungiSMASH detected 13 693 BGCs in 373 ingroup taxa, of these 6129 belonged to LFF (136 species) and 7558 to nLFF (237 species) (Fig. 1). The total number of BGCs is displayed on the phylogenomic tree using colored bars, whereas the number of PKS, NRPS, terpene, and RiPP BGCs is displayed as heatmaps. Between the groups LFF and nLFF, LFF harbor a significantly higher number of BGCs as compared to the nLFF (intercept 45.066, β_1_ estimate −13.26, *P*‐value < 0.01). LFF exhibit significantly higher numbers of PKS gene clusters (intercept 18.6912, β_1_ estimate −8.7327, *P*‐value <0.001). In the randomized analysis, with a sample size of 100 and 10 000 iterations, all iterations were found to be significant, supporting that LFF have a significantly higher number of total BGCs and PKS clusters than nLFF despite the taxa choice. Non‐lichenized fungi, by contrast, demonstrate a pronounced enrichment in NRPS BGCs, identified in 85% of the iterations. In comparison, BGCs associated with terpene and RiPP synthesis did not show a strong or consistent enrichment in either LFF or nLFF, with 51% and 61% of iterations, respectively, favoring LFF as enriched. This suggests that these BGC classes may not be strongly correlated with lifestyle.

## Discussion

In this study, we test whether LFF are a treasure chest of natural products, by mining the genomes of LFF and nLFF in Pezizomycotina (Ascomycota). We used BGC count as a proxy for biosynthetic potential.

We found that LFF possess a significantly higher number of BGCs than nLFF, providing statistical evidence to support this long‐standing concept. Interestingly, while the total number of BGCs is generally higher in LFF than in nLFF, this effect is primarily driven by the PKS clusters. This is consistent with the remarkable diversity of PKS metabolites produced by LFF (Boustie & Grube, 2005; Goga *et al*., 2018). Conversely, nLFF genomes contain a higher number of NRPS and PKS–NRPS hybrid BGCs. Since the primary distinction between these two groups is that LFF exist in an obligate symbiotic association with algae, it is compelling to suggest that the long‐term evolutionary relationship with algae may have driven the expansion of metabolism‐related gene families in LFF.

A recent study investigating major evolutionary changes between fungi and animals revealed expansions in carbohydrate and secondary metabolism‐related gene families in fungi (Ocaña‐Pallarès *et al*., 2022), underscoring the fundamental role of secondary metabolism in fungal evolution. This expansion of genes families related to secondary metabolism is especially pronounced in symbiotic fungi, such as in LFF, where complex associations seem to have driven further diversification of metabolic pathways. Notably, the lichenized lifestyle involves several steps that require an interplay of metabolic interactions between symbionts – for instance, lichenization (reformation of the symbiotic association between the LFF and the photobiont) itself comprises various steps including partner recognition and contact establishment (Pichler *et al*., 2023). It is thus reasonable to infer that organisms involved in complex relationships evolve a sophisticated array of mechanisms to enable metabolic cross talk between symbionts.

Experimental evidence reinforces this effect as coculturing fungi with other microorganisms activates numerous BGCs, including even the silent ones (Wang *et al*., 2023; Xu *et al*., 2023). For example, in experiments involving *Heterobasidion annosum*, *Gloeophyllum sepiarium*, and *Armillaria ostoyae* (Xu *et al*., 2023), the presence of other fungi triggered an increase of up to 400‐fold in secondary metabolite production, underscoring how biotic interactions can enhance metabolic output in fungi. Similarly, cocultivating *Aspergillus nidulans* with a collection of soil bacteria activated secondary metabolism genes in the fungus (Schroeckh *et al*., 2009). This study showed that intimate physical interaction between the bacterial and fungal mycelia was essential for eliciting the response. Interestingly, one of the BGCs activated by the cocultivation codes for the polyketide orsellinic acid, which is similar to, or an intermediate of, the metabolite lecanoric acid, secreted by several LFF. This further suggests that the long evolutionary relationship between bacteria, algae, and fungi may have contributed to the high metabolic potential observed in LFF. Furthermore, the metabolic profile of mycobiont grown in isolation is different from that of the lichen (Cordeiro *et al*., 2004; Alors *et al*., 2023), highlighting the influence of biotic associations on the secondary metabolite profile of lichens.

Furthermore, several recent studies suggest that lichens are complex ecosystems hosting a plethora of other organisms, including yeast and bacteria (Grube & Spribille, 2012; Spribille *et al*., 2016; Allen & Lendemer, 2022; Cometto *et al*., 2022). Although we do not currently know the levels of interdependence and specificity of some of the organisms in lichen thalli, these associations may have served as an evolutionary engine for metabolite diversification, enriching the metabolic ‘treasure’ of lichens. In fact, variations in lichen metabolite profiles appear to be influenced by the algal partner, with distinct BGCs activated when associating with different alga (Farkas *et al*., 2024). This suggests that the algal partner plays a role in shaping the metabolic profile of LFF.

Beyond the role of symbiosis, lichens exhibit an extraordinary adaptability to diverse ecological niches, from arctic tundras to desert landscapes. This ecological success, in some cases, could be due to their ability to switch between different algal partners (e.g. Dal Grande *et al*., 2017), but recent evidence suggests that chemical diversity also plays a critical role (Schweiger *et al*., 2022). Studies on *Umbilicaria* have shown climate‐driven BGC variation, indicating that environmental conditions can act as triggers for unique biosynthetic outputs (Singh *et al*., 2021).

Despite the high biosynthetic potential of LFF, the pharmaceutical utilization of their metabolites is negligible due to the complex lifestyle, which presents significant challenges for metabolite production under controlled conditions. For instance, the slow growth rates of LFF in culture may place them at a disadvantage compared to nLFF when it comes to producing useful metabolites *in vitro*. However, these obstacles may soon be overcome, as several protocols for the successful culturing of LFF and the heterologous expression of their metabolites are already available (Kealey *et al*., 2021; Kim *et al*., 2021; Singh, 2023; Rosabal & Pino‐Bodas, 2024).

In conclusion, we demonstrate the higher biosynthetic potential of LFF as compared to nLFF and propose that the remarkable biosynthetic diversity observed in lichens arises from a combination of intricate biotic interactions and exceptional ecological adaptability. Specifically, the symbiotic relationship between the fungus and its algal partner, along with interactions with other microbial inhabitants within the lichen thallus, creates a dynamic metabolic environment. This environment may have driven the evolution of metabolic versatility in these organisms, making them a treasure chest of bioactive metabolites. Given these factors, we advocate for the utility of coculturing studies to activate silent BGCs in lichens. Since the enrichment of BGCs in LFF is primarily driven by PKS clusters, future studies should focus on comparing the evolutionary history of PKS clusters between LFF and nLFF.

## Competing interests

None declared.

## Author contributions

GS planned and designed the research. GS and AP gathered and analyzed data. GS and AP wrote the manuscript. Both authors have read and approved the final version of the manuscript.

## Supporting information


**Notes S1** Statistical analyses implemented in the study.

**Table S1** Voucher information of the taxa used in the study.
Please note: Wiley is not responsible for the content or functionality of any Supporting Information supplied by the authors. Any queries (other than missing material) should be directed to the *New Phytologist* Central Office.
