# Supplementary material for: Lichens are a treasure chest of bioactive compounds: fact or fake?
Source: New Phytol. 2025 Feb 27;246(2):389–95. doi: 10.1111/nph.70034 (PMC11923404; doi:10.1111/nph.70034)
Supplement: Supplementary file 1 — Notes S1 Statistical analyses implemented in the study. [file NPH-246-389-s002.docx]

## *New Phytologist* Supporting Information

Article title: Lichens are a treasure chest of bioactive compounds: Fact or fake?

Authors: Anna Pasinato, Garima Singh

Article acceptance date: 05 February 2025

The following Supporting Information is available for this article:

**Table S1** Voucher information of the taxa used in the study including the genome assembly and biosynthetic gene statistics

**Provided separately**

**Notes S1** Statistical analyses implemented in the study.

**#######Significance Test**

Call:

lm(formula = `BGCs total` ~ Lifestyle)

Residuals:

Min 1Q Median 3Q Max

-40.066 -14.066 -3.066 12.199 97.199

Coefficients:

Estimate Std. Error t value Pr(>|t|)

(Intercept) 45.066 1.740 25.896 < 2e-16 ***

Lifestylenon-lichen -13.265 2.177 -6.094 2.73e-09 ***

---

Signif. codes: 0 ‘***’ 0.001 ‘**’ 0.01 ‘*’ 0.05 ‘.’ 0.1 ‘ ’ 1

Residual standard error: 20.3 on 375 degrees of freedom

Multiple R-squared: 0.09012, Adjusted R-squared: 0.08769

F-statistic: 37.14 on 1 and 375 DF, p-value: 2.728e-09

Analysis of Variance Table

Response: BGCs total

Df Sum Sq Mean Sq F value Pr(>F)

Lifestyle 1 15002 15001.6 36.116 4.445e-09 ***

Residuals 371 154104 415.4

---

Signif. codes: 0 ‘***’ 0.001 ‘**’ 0.01 ‘*’ 0.05 ‘.’ 0.1 ‘ ’ 1

--------------------------------------------------------

Call:

lm(formula = PKSs ~ Lifestyle)

Residuals:

Min 1Q Median 3Q Max

-18.691 -5.959 -1.959 5.041 43.041

Coefficients:

Estimate Std. Error t value Pr(>|t|)

(Intercept) 18.6912 0.7720 24.212 <2e-16 ***

Lifestylenon-lichen -8.7327 0.9655 -9.045 <2e-16 ***

---

Signif. codes: 0 ‘***’ 0.001 ‘**’ 0.01 ‘*’ 0.05 ‘.’ 0.1 ‘ ’ 1

Residual standard error: 9.003 on 375 degrees of freedom

Multiple R-squared: 0.1791, Adjusted R-squared: 0.1769

F-statistic: 81.8 on 1 and 375 DF, p-value: < 2.2e-16

Analysis of Variance Table

Response: PKSs

Df Sum Sq Mean Sq F value Pr(>F)

Lifestyle 1 6540 6540.0 79.895 < 2.2e-16 ***

Residuals 371 30369 81.9

---

Signif. codes: 0 ‘***’ 0.001 ‘**’ 0.01 ‘*’ 0.05 ‘.’ 0.1 ‘ ’ 1

--------------------------------------

Call:

lm(formula = NRPs ~ Lifestyle)

Residuals:

Min 1Q Median 3Q Max

-12.0515 -5.0515 -0.9502 3.9485 29.0498

Coefficients:

Estimate Std. Error t value Pr(>|t|)

(Intercept) 13.0515 0.6136 21.270 <2e-16 ***

Lifestylenon-lichen -1.1013 0.7675 -1.435 0.152

---

Signif. codes: 0 ‘***’ 0.001 ‘**’ 0.01 ‘*’ 0.05 ‘.’ 0.1 ‘ ’ 1

Residual standard error: 7.156 on 375 degrees of freedom

Multiple R-squared: 0.005461, Adjusted R-squared: 0.002809

F-statistic: 2.059 on 1 and 375 DF, p-value: 0.1521

Analysis of Variance Table

Response: NRPSs

Df Sum Sq Mean Sq F value Pr(>F)

Lifestyle 1 98.6 98.629 1.9125 0.1675

Residuals 371 19132.6 51.570

---------------------------------------------------

Call:

lm(formula = Terpenes ~ Lifestyle)

Residuals:

Min 1Q Median 3Q Max

-5.4706 -2.3651 -0.4706 1.5294 21.6349

Coefficients:

Estimate Std. Error t value Pr(>|t|)

(Intercept) 6.4706 0.3057 21.167 < 2e-16 ***

Lifestylenon-lichen -1.1054 0.3823 -2.891 0.00406 **

---

Signif. codes: 0 ‘***’ 0.001 ‘**’ 0.01 ‘*’ 0.05 ‘.’ 0.1 ‘ ’ 1

Residual standard error: 3.565 on 375 degrees of freedom

Multiple R-squared: 0.02181, Adjusted R-squared: 0.0192

F-statistic: 8.36 on 1 and 375 DF, p-value: 0.00406

Analysis of Variance Table

Response: Terpenes

Df Sum Sq Mean Sq F value Pr(>F)

Lifestyle 1 100.5 100.45 7.842 0.005372 **

Residuals 371 4752.4 12.81

---

Signif. codes: 0 ‘***’ 0.001 ‘**’ 0.01 ‘*’ 0.05 ‘.’ 0.1 ‘ ’ 1

-----------------------------------------------------

Call:

lm(formula = RiPPs ~ Lifestyle)

Residuals:

Min 1Q Median 3Q Max

-7.963 -4.963 -1.963 3.008 32.008

Coefficients:

Estimate Std. Error t value Pr(>|t|)

(Intercept) 7.9632 0.5510 14.452 < 2e-16 ***

Lifestylenon-lichen -1.9715 0.6892 -2.861 0.00446 **

---

Signif. codes: 0 ‘***’ 0.001 ‘**’ 0.01 ‘*’ 0.05 ‘.’ 0.1 ‘ ’ 1

Residual standard error: 6.426 on 375 degrees of freedom

Multiple R-squared: 0.02136, Adjusted R-squared: 0.01875

F-statistic: 8.184 on 1 and 375 DF, p-value: 0.004464

Analysis of Variance Table

Response: RiPPs

Df Sum Sq Mean Sq F value Pr(>F)

Lifestyle 1 335.9 335.93 8.0851 0.00471 **

Residuals 371 15414.8 41.55

---

Signif. codes: 0 ‘***’ 0.001 ‘**’ 0.01 ‘*’ 0.05 ‘.’ 0.1 ‘ ’ 1

--------------------------------------------------

Call:

lm(formula = Hybrid ~ Lifestyle)

Residuals:

Min 1Q Median 3Q Max

-1.3235 -1.2822 -0.3235 0.7178 6.7178

Coefficients:

Estimate Std. Error t value Pr(>|t|)

(Intercept) 1.32353 0.12685 10.434 <2e-16 ***

Lifestylenon-lichen -0.04137 0.15865 -0.261 0.794

---

Signif. codes: 0 ‘***’ 0.001 ‘**’ 0.01 ‘*’ 0.05 ‘.’ 0.1 ‘ ’ 1

Residual standard error: 1.479 on 375 degrees of freedom

Multiple R-squared: 0.0001813, Adjusted R-squared: -0.002485

F-statistic: 0.068 on 1 and 375 DF, p-value: 0.7944

Analysis of Variance Table

Response: Hybrid

Df Sum Sq Mean Sq F value Pr(>F)

Lifestyle 1 0.12 0.11582 0.0526 0.8187

Residuals 371 816.25 2.20015

**#########Randomisation results**

**Total BGCs

Significant iterations: 10000

Total iterations: 10000

Proportion significant: 1

**PKS

Significant iterations: 10000

Total iterations: 10000

Proportion significant: 1

**NRPS

Significant iterations: 1584

Total iterations: 10000

Proportion significant: 0.1584

**Terpenes

Significant iterations: 6178

Total iterations: 10000

Proportion significant: 0.6178

**RiPPs

Significant iterations: 5668

Total iterations: 10000

Proportion significant: 0.5668

**hybrid BGCs

Significant iterations: 60

Total iterations: 10000

Proportion significant: 0.006

**#########Genome quality assessment**

Pearson's product-moment correlation

data: Genome_size and BGCs

t = 4.1068, df = 398, p-value = 4.871e-05

alternative hypothesis: true correlation is not equal to 0

95 percent confidence interval:

0.1056650 0.2938697

sample estimates:

cor

0.201627

Pearson's product-moment correlation

data: n_Scaffolds and BGCs

t = 4.4081, df = 399, p-value = 1.342e-05

alternative hypothesis: true correlation is not equal to 0

95 percent confidence interval:

0.1201028 0.3069491

sample estimates:

cor

0.2154975
